# Correlation of language lateralization with resting state hippocampal connectivity in temporal lobe epilepsy patients

**DOI:** 10.3906/sag-2001-29

**Published:** 2020-08-26

**Authors:** Ali Murat KOÇ, Ali Yusuf ÖNER, Halil ÖZER, Melike GÜRYILDIRIM, Emin Turgut TALI, Fatih ÖNCÜ, Murat UÇAR, Erhan BİLİR, İrem ÇAPRAZ, Gökhan KURT

**Affiliations:** 1 Department of Radiology, School of Medicine, Gazi University, Ankara Turkey; 2 Department of Neurology, School of Medicine, Gazi University, Ankara Turkey; 3 Department of Neurosurgery, School of Medicine, Gazi University, Ankara Turkey

**Keywords:** Functional magnetic resonance imaging, hippocampus, language, epilepsy

## Abstract

**Background/aim:**

The main purpose of this study is to evaluate the resting state hippocampal connectivity with language areas and to correlate them with laterality index calculations on single subject basis, hence to present hippocampal lateralization for language with rs-fMRI.

**Materials and methods:**

Task based and rs-fMRI data were gathered from a total of 45 subjects in 3T scanner. BrainVoyager QX, SPM, and CONN softwares were used for data analysis. LI score of each subject was calculated and converted into normalized LI score (nLI). Intrahemispheric rs-connectivity analysis was performed between hippocampus and Broca’s regions on both sides. Correlation between these variables was measured with SPSS software.

**Results:**

Right-TLE patients were found to have highest whereas left-TLE group were found to have lowest mean LI scores. Regarding hippocampal-lingual networks; left intrahemispheric connectivity values showed strong positive correlation with nLI values in left, right-TLE patients and healthy controls (P = 0.035, 0.014, 0.047). There were no significant correlation between right intrahemispheric connectivity values and nLI scores in all groups.

**Conclusion:**

This study seems to depict the existence of resting state hippocampal-lingual functional network which correlates well with lateralization of language function in the left hemisphere in both temporal lobe epilepsy patients and healthy controls.

## 1. Introduction

Epilepsy is a chronical disease affecting language and other cognitive functions [1]. Approximately 10%–30% of patients with idiopathic epilepsy or hippocampal sclerosis as mesial temporal lobe epilepsy (MTLE) are medically refractory to antiepileptic drugs (AED) and require surgical treatment aimed to remove hippocampus, amygdala and anterior temporal lobe [2]. Outcomes of epilepsy surgery are good, and are better in TLE, providing a seizure free life in most of the patients [3]. Although overwhelming majority of the right-handed population (95%) have left hemispheric dominancy; the higher possibility of atypical dominancy in TLE patients [4] ultimately rises the need for precise determination of hemispheric dominancy for language and memory functions to avoid any possible cognitive deficit after surgical treatment [5,6].

Magnetic resonance imaging (MRI) is the best imaging modality in epilepsy [2]. Mapping of eloquent cortical networks with functional MRI (fMRI) reveals the chance for surgical treatment and reduces the possibility of postoperative neurological deficits (i.e. speech or visual loss) by demonstrating the anatomical relation between epileptogenic foci and functionally activated areas [7]. Conventional task based (tb) fMRI has the capability of defining regions that are anatomically connected and activated during a given task of interest; whereas resting-state (rs) fMRI maps functional connections with their strength and direction by analyzing the baseline spontaneous fluctuations of blood oxygenation level dependent (BOLD) signals throughout the brain, resulting in several cognitive networks [1, 8–10]. Laterality index (LI) is an indirect marker for hemispheric dominancy of language and can be calculated from the data driven with tb-fMRI. 

Studies of unilateral and bilateral hippocampal damage have shown that left hippocampus was found to have stronger relation with language processing whereas right hippocampus has mostly related with visuospatial processes [11,12]. Hippocampus is thought to have an indirect relation with language networks; it plays role in establishing early intrahemispheric connections rather than a direct connectivity with language areas. Since degenerative processes in TLE patients cause the hippocampus at the effected side to lose neurons and became atrophied, it can be hypothesised that its direct or indirect functional connectivity with other brain regions, preferentially ipsilateral language areas, is decreased compared to healthy subjects.

The main purpose of this study is to evaluate whether the resting state intrahemispheric hippocampal connectivity with language areas correlates with laterality indices on single subject basis. Furthermore, we aimed to find out if there is a hippocampal lateralization for language. To the best of our knowledge, this is the first study correlating hippocampal-lingual resting state functional connectivity with hemispheric lateralization for language. 

## 2. Materials and methods 

### 2.1. Patient selection

This study is approved by the institutional ethics committe (decision number: 25901600-958). Written informed consent was received from the participants. Patients who have admitted to outpatient clinics with symptoms of multiple seizures were clinically evaluated in neurology and neurosurgery departments by two neurologists with 7 and 24 years of experience in epilepsy (IYC, EB) and one neurosurgeon with 11 years of experience in epilepsy surgery (GK). Among those, the group of patients who have diagnosed to have intractable epilepsy that is medically refractory to AEDs were further evaluated with video-EEG, neurocognitive tests, cranial MRI with dedicated epilepsy protocol, functional MRI, MR-spectroscopy (MRS), MR-perfusion (MRP) and interictal PET-CT imaging as a routine presurgical workup. This study focuses on fMRI examination of these group of epilepsy patients. Processing, analysis and evaluation of fMRI and cranial MRI were performed by two neuroradiologist with 6 and 29 years of experience (AYO, ETT) and five radiologists (AMK, HO, MG, FO, MU) in section of neuroradiology.

Thirty mesial temporal lobe epilepsy patients with medically intractable epileptic seizures who were selected candidates for epilepsy surgery and fifteen healthy control subjects were included to the study. Patients who have neurological or psychiatric diseases other than epilepsy, non-Turkish primary language, extratemporal or bitemporal source of epilepsy, history of cranial surgery or nonepileptic cranial insult, left hand dominancy, known contraindications for MRI examinations were not included to the study. Control subjects were assessed only with cranial MRI before fMRI workup who were clinically examined and proven to be neurologically healthy. All subjects were right handed according to the Edinburgh Handedness Inventory [13]. Patients were grouped as right and left-TLE according to outcomes of pre-surgical workup with consensus of all departments included in study (Table 1).

**Table 1:** Demographic data, presurgical workup results and side of epilepsy.

Subject No.	Age	Sex	Age at seizure onset	Seizure duration^a^	Structural MRI^b^	MRS	MRP	PET-CT	EEG	Side of epilepsy^c^	Childhood medical history
1	43	FM	20	23	Right HA-S	N	N	Right	Bilateral	Right	
2	29	FM	12	17	Right HA-S	Right	N	Right	Right	Right	Head trauma
3	22	FM	13	9	Right HA-S	Right	N	Right	Normal	Right	
4	26	FM	20	6	Normal	N	N	Right	Normal	Right	
5	39	M	16	23	Right HA-S	Right	Right	Right	Right	Right	Febrile convulsion
6	19	FM	7	12	Normal	N	N	N	Right	Right	Febrile convulsion
7	27	FM	22	5	Left HA-S	Bilateral	Bilateral	Right	Right	Right	-
8	49	FM	35	14	Normal	Normal	Normal	Right	Right	Right	-
9	34	M	17	17	Right HA-S	Right	Right	Right	Right	Right	-
10	18	M	15	3	Right HA-S	Right	Right	Right	Right	Right	-
11	41	M	22	19	Left HA-S	N	Left	N	Left	Left	-
12	38	FM	7	31	Left HA-S	Left	Left	Left	Left	Left	Febrile convulsion
13	22	FM	1	21	Left HA-S	Left	Left	Left	Left	Left	Head trauma
14	23	FM	19	4	Normal	Left	N	Left	Left	Left	-
15	21	M	11	10	Left HA-S	Left	N	Left	Left	Left	Head trauma
16	29	FM	8	21	Left HA-S	Left	Left	Left	Left	Left	Febrile convulsion
17	18	FM	2	16	Left HA-S	Left	Normal	Left	Normal	Left	-
18	33	M	16	17	Left HA-S	Left	Normal	Left	Left	Left	-
19	29	M	1	28	Left HA-S	Left	Left	Left	Left	Left	Febrile convulsion
20	35	FM	31	4	Normal	N	N	Left	N	Left	-
21	31	FM	1	30	Left HA-S	Left	N	Left	Left	Left	-
22	37	FM	35	2	Left HA-S	Left	Left	Left	Left	Left	Head trauma
23	33	FM	-	-	Normal	-	-	-	-	-	-
24	29	FM	-	-	Normal	-	-	-	-	-	-
25	31	FM	-	-	Normal	-	-	-	-	-	-
26	26	M	-	-	Normal	-	-	-	-	-	-
27	44	FM	-	-	Normal	-	-	-	-	-	-
28	28	M	-	-	Normal	-	-	-	-	-	-
29	26	M	-	-	Normal	-	-	-	-	-	-
30	31	FM	-	-	Normal	-	-	-	-	-	-
31	31	M	-	-	Normal	-	-	-	-	-	-
32	27	FM	-	-	Normal	-	-	-	-	-	-

HA-S: Hippocampal atrophy and sclerosis; MRS: MR-Spectroscopy; MRP: MR-Perfusion; Right, Left, N (normal), Bilateral: Side of pathology. ^a^Seizure duration, years; ^b^Structural MRI refers to dedicated epilepsy protocol; ^c^Side of epilepsy were decided by multi-department consensus according to outcomes of presurgical workup.

### 2.2. MRI examination

All subjects were examined with an 8-channel head coil on a 3T MRI scanner (Siemens Magnetom Verio, Siemens AG, Erlangen, Germany). All patients were first scanned with dedicated epilepsy protocol (Supplement 1). Functional MR imaging protocol was composed of resting state (TE: 30 ms; TR: 2000 ms; flip angle: 90; slice thickness: 3 mm; matrix: 64x64; FOV: 235; 27 slices) and task based (TE: 30 ms; TR: 3000 ms; flip angle: 90; slice thickness: 4 mm; matrix: 64x64; FOV: 235; 30 slices) functional MR images which were acquired consecutively. Echoplanar imaging (EPI) with T2* gradient echo sequences were used for fMRI examinations. All patients were scanned during their interictal period. Tasks were given with an MRI-compatible LCD screen which is visible through a small mirror attached to the head coil. LCD screen was connected to a computer at the operating room (Functional MRI Imaging Equipments, Telemed Ltd., İstanbul, Turkey). The stimuli were presented electronically using the E-Prime 2.0 software (Psychology Software Tools, Inc., Sharpsburg, PA, USA) [14]. Subjects also wore over-ear headphones connected to the MRI console to lower the background noise and to communicate if needed. Rs- and tb-fMRI paradigms were run once for each patient. Total duration of MRI examination per subject was 13 min and 4 s.

#### 2.3.1. FMRI paradigm 

##### 2.2.1.1. Resting state fMRI

Subjects did not perform any task but they were prompted to not to think anything specific. Since we had experienced in our institution that the patients were usually prone to sleep when their eyes are closed during resting state fMRI examinations, all subjects were warned to keep their eyes open and fixed. First 20 seconds of paradigm was spared as dummy and eventually excluded from data in preprocessing steps to overcome T1-saturation effect. Total runtime was 368 s.

##### 2.2.1.2. Task based fMRI

**Figure 1:**
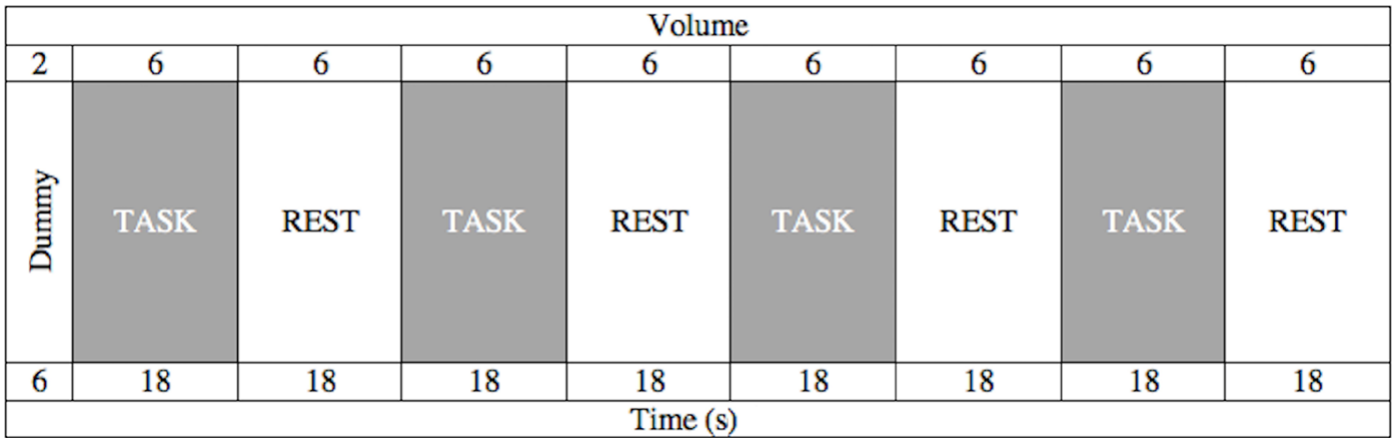
Diagram showing word generation task - fMRI paradigm.

A block designed word generation task was used. All subjects were trained with a short introductory task on computer before MRI examination to familiarize themselves with the task to be performed. Subjects were prompted to follow the on-screen instructions and do the given task silently during MRI examination. First 6 s were spared as a dummy similar to the rs-fMRI paradigm. 6 different words were displayed during a stimulation (task) block that’s followed by a resting (rest) block with same duration, 18 s each. The task block was composed of mixed words appearing with predefined duration and time intervals. Subjects were expected to quickly generate verbs relevant to the on-screen appearing words (nouns). Rest blocks consist of a constant sign (plus sign: “+”) that tells subjects to rest and don’t think anything in particular. Four pairs of separate task and rest blocks, one dummy block and 8 s required for sequence preparation makes a total of 158 s run time (Figure 1).

### 2.3. Data analysis

Preprocessing and statistical analysis of tb-fMRI data were performed with BrainVoyager Software (Version 20.0, Brain Innovation BV, Maastricht, Netherlands) [15]. Preprocessing of resting state data was performed with *SPM* (Statistical Parametric Mapping, Version 8, Wellcome Department of Cognitive Neurology,) and functional connectivity analysis (FC) was performed with *CONN* (Functional Connectivity Toolbox for SPM) softwares which both require *MATLAB* (MATLAB and Statistics Toolbox Release R2015a, The MathWorks, Inc., Natick, MA, USA) to run. Further statistical analyses were performed with *SPSS* software (SPSS for Windows, Version 15.0. SPSS Inc., Chicago, IL, USA). 

#### 2.3.1. Tb-fMRI preprocessing & analysis

High resolution T1-weighted anatomical images were used to define the precise cerebral parenchymal borders using 9-parameter marking system. After manual definition of anterior and posterior commissures, subjects’ anatomic data were normalized to Talairach space. After than, the following preprocessing steps were applied to the functional data consequtively; mean intensity adjustment, slice-timing correction, 3D-head motion correction, spatial smoothing (Gaussian filter, full width half maximum: 4 mm) and temporal filtering (high pass filter: 2sin/cos). Patients who had moved their head more than 2 mm in any of 6 axis during the scan were excluded from the study (one patient from each TLE groups and two subjects from healthy control group). fMRI data was than normalized to Talairach space and coregistered with T1 anatomical data. Further normalization into 3D space generated a volume-time course (VTC) data. After application of hemodynamic response function (HRF) correction, General linear modeling (GLM) analysis was applied to this VTC data. Resulting single subject activation map showed multiple activated voxels across the whole brain. Since EPI sequence is highly sensitive and vulnerable to artifacts, we used a cluster threshold and cluster extent of voxel threshold at a default level defined by the software vendor (50 voxels and 4 contiguous voxels) and the statistical threshold was set at P < 0.05. Negative activations were removed, total number of activated voxels were labeled as Vtotal and recorded for each subject. Regions of interests (ROI) were than defined one for each Broca’s area (Brodmann’s areas 44 and 45) via BrainVoyager’s template of Brodmann’s areas. 

#### 2.3.2. Calculation of laterality index and normalized laterality index

Number of activated voxels within right (Vright) and left ROIs (Vleft) were recorded from task based fMRI data. Laterality Index was then calculated for each subject with formula given in Table 2 [16]. LI calculation yielded scores between values of +100 and –100 indicating strong left and strong right hemispheric dominancy respectively. Next, percentage of activated voxels within predefined language areas represented in the whole brain were calculated (Plang, Table 2). Reproducibility of fMRI had already been tested in various studies [17–20]. Although results of same fMRI studies applied to same patients at different times are expected to be highly accurate (hemispheric dominancy, i.e.), precise number of activated voxels may not be identical due to excessivity of factors influencing the BOLD activity. To overcome this problem and to compare different subjects more efficiently, we assumed that the percentage of activated voxels related to language activity within the whole brain rather stays the same during a verb generation task and hence defined the *normalized* laterality index (nLI, Table 2). Since nLI scoring is directly dependent to Plang values, it does not have strict borders like LI; theoretical upper and lower limits are indefinite. 

**Table 2:** Task based fMRI results, lateralization index calculations of all subjects and side of epilepsy in TLE patients.

Subject No.	P_lang_^a^	LI^b^	nLI^c^	Language lateralization^d^	Side of epilepsy
1	1.830	59	32	Typical	Right-TLE
2	1.878	94	50	Typical	Right-TLE
3	0.987	46	47	Typical	Right-TLE
4	1.610	76	47	Typical	Right-TLE
5	1.755	21	12	Typical	Right-TLE
6	0.876	85	97	Typical	Right-TLE
7	0.896	50	56	Typical	Right-TLE
8	1.189	53	44	Typical	Right-TLE
9	1.351	5	4	Atypical^e^	Right-TLE^f^
10	1.322	41	31	Typical	Right-TLE
11	2.614	94	36	Typical	Left-TLE^f^
12	1.365	9	6	Atypical^e^	Left-TLE
13	0.192	39	202	Typical	Left-TLE^f^
14	0.712	85	119	Typical	Left-TLE^f^
15	1.609	56	35	Typical	Left-TLE^f^
16	0.385	85	221	Typical	Left-TLE^f^
17	0.968	63	65	Typical	Left-TLE^f^
18	0.826	–18	–21	Atypical^e^	Left-TLE
19	1.070	–33	–31	Atypical^e^	Left-TLE
20	0.735	50	68	Typical	Left-TLE^f^
21	1.142	–18	–16	Atypical^e^	Left-TLE
22	0.594	–51	–86	Atypical^e^	Left-TLE
23	1.317	69	53	Typical	-
24	2.347	26	11	Typical	-
25	0.641	16	25	Typical	-
26	1.140	56	49	Typical	-
27	1.191	44	37	Typical	-
28	2.190	4	2	Atypical^e^	-
29	2.363	6	3	Atypical^e^	-
30	3.114	94	30	Typical	-
31	1.080	30	28	Typical	-
32	1.270	90	71	Typical	-

V_left_ and V_right_ below refers to number of activated voxels within Broca’s regions of both hemispheres, Vtotal refers to total number of activated voxels within whole brain. ^a^Percentage of activated voxels within predefined language areas, P_lang_= (V_left_ +V_right_)/V_total_; ^b^Lateralization index, LI = (V_left_ -V_right_)*100/(V_left_ +V_right_); ^c^Normalized lateralizaton index, nLI = (LI/P_lang_)*100; ^d^Language lateralizations were calculated according to LI scores; ^e^Subjects with atypical hemispheric dominancy for language (right or bilateral dominancy) according to LI scores; ^f^Patients who have increased risk for possible postoperative language deficits.

#### 2.3.3. Rs-fMRI preprocessing and fc analysis

Resting state functional data was first realigned, coregistered with anatomical data, segmented, normalized, and smoothed (Gaussian smoothing, FWHM: 9 mm) in SPM’s preprocessing step. Then, white matter, CSF signals and 6-plane rigid body motion parameters were removed from BOLD data with component-based noise correction method (CompCor) in CONN toolbox [21]. Subjects with major artifacts, such as excessive head motions, were excluded from the study (four patients from right-, two patients from left-TLE and three subjects from healthy control group). A low frequency filter was used in denoising step to eliminate physiological noise originating from cardiac and respiratory cycles (0.008–0.9 Hz). ROI to ROI 2nd level analysis was chosen in the analysis step: bilateral IFG, pars opercularis and pars triangularis were selected as target; bilateral hippocampi were selected as seed ROIs. All target and seed ROIs were predefined in CONN tolbox, SPM. FC analysis were than performed for both hemispheres with correlation of time courses of ipsilateral seed and target ROIs. Beta values (FC values) of each subject contributing to the strength of group functional connectivity were extracted.

### 2.4. Statistical analysis

Variables were investigated using visual (histograms, probability plots) and analytical methods (Shapiro–Wilk test) to determine whether or not they were normally distributed. The Mann–Whitney U test was used to compare the age at seizure onset and duration of the seizure between left-TLE and right-TLE patients. Kruskal–Wallis tests were conducted to compare nonnormally distributed variables and one-way ANOVA test were used to compare normally distributed variables among left-TLE, right-TLE patients and control groups. FC values of the both seed ROIs were analyzed with one-way ANOVA design to find out potential differences among groups. nLI values, which were formerly obtained from tb-fMRI examination, and FC values of each subject were selected as independent and dependent variables respectively. Spearman test was used for nonnormally distributed and Pearson test was used for normally distributed variables.

## 3. Results

Fifteen patients with right sided TLE were classified as “right-TLE”; 15 patients with left sided TLE were classified as “left-TLE” groups. Average onset of seizures was 14.47 and duration of epilepsy was 15.83 years (18.73 and 10.2 in right-TLE; 13.07 and 18.6 in left-TLE groups respectively). Mean age of all patients was calculated as 30.28 ± 7.7 years. Some of the subjects were excluded from the study after preprocessing steps (see above) resulting in a total of 32 subjects (10 patients in right-TLE, 12 patients in left-TLE groups; 10 subjects in healthy controls; a total of 21 female, 11 male).

**Figure 2:**
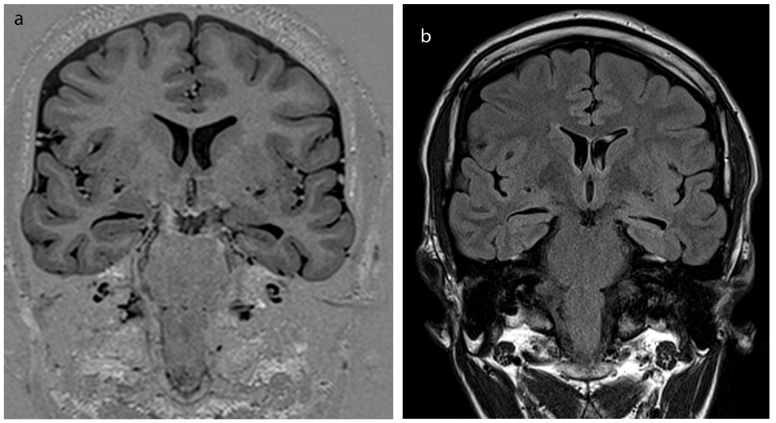
(A) High resolution epilepsy dedicated imaging of subject no. 19. Left hippocampal atrophy can be depicted from isotropic, coronal oblique T1-IR sequence. (B) FLAIR image also shows increased signal at the side of atrophy, revealing hippocampal sclerosis of the same subject.

There were no significant differences in age between controls versus right TLE patients (F = 3.16; P = 0.10), controls versus left TLE patients (F = 0.34; P =0.57) nor between TLE groups (F = 1.05; P = 0.32). Moreover, patient groups showed no statistically significant difference in terms of the age at seizure onset (Mann–Whitney U test; P = 0.186) and seizure duration (Mann–Whitney U test; P = 0.390). Mean onset of seizures found as 14.2 ± 9.2 years ranging from 1 to 17 years, and duration of epilepsy was found as 15.3 ± 8.7 years in TLE patients. Seven patients in right-TLE and ten patients in left-TLE group were found to have hippocampal atrophy and sclerosis (70%; 83%) on structural MRI examinations (Figures 2A and 2B). Remaining five patients did also show pathology on MRS, MRP, PET-CT and EEG. Subjects’ demographic data, childhood medical history, structural MRI, presurgical workup results and lateralization in TLE patients are shown in Table 1.

### 3.1. Analysis of task based-fMRI and laterality indices

**Figure 3:**
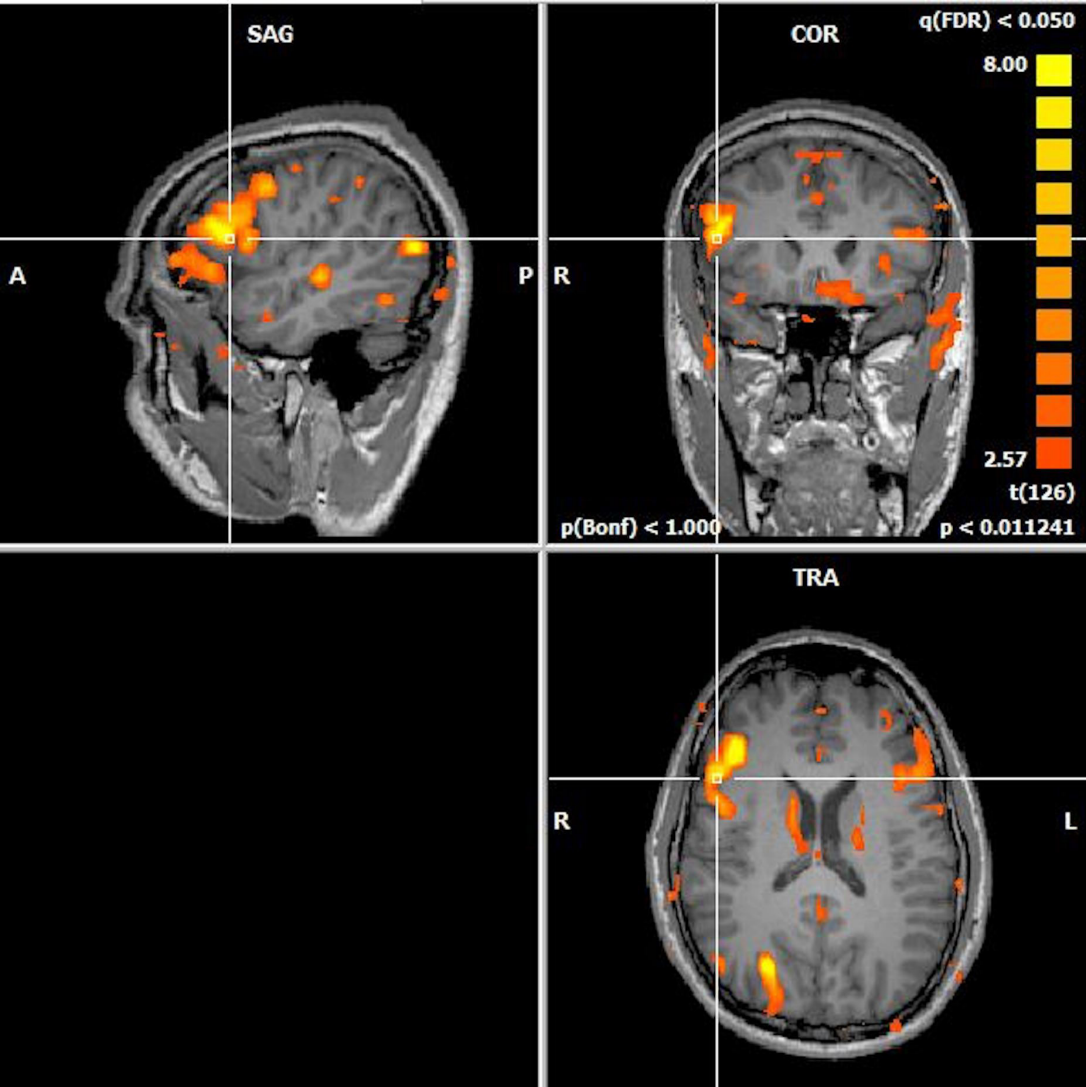
(Activation map generated from task-based fMRI data of a subject with left hippocampal atrophy. Asymmetrical, atypical right dominant activation at IFG, Broca’s regions are shown (BrainVoyager QX).

Tb-fMRI examination successfully yielded number of activated voxels at both Broca’s regions (Vright and Vleft) and whole brain (Vtotal) in each subject (Figure 3). There was no statistically significant difference between groups regarding number of activated voxels in whole brain and percentage of language areas within whole brain (Plang). Based on these, LIs were calculated. Patients with LI scores ≥10 were accepted to have typical (left) hemispheric dominancy for language whereas patients with LI scores <10 were accepted to have atypical dominancy (right or bilateral) [22]. One patient from right-TLE (10%), five patients from left-TLE (41.7%) and two subjects from healthy control group (20%) were found to have atypical language dominancy according to LI scores. Right-TLE patients were found to have highest and left-TLE group were found to have lowest mean LI scores (right-TLE: 53; left-TLE: 30; control group: 43). But, mean laterality index values of groups did not show statistically significant difference among three groups (P = 0.582). nLIs were basically calculated from LI scores and voxelwise percentage of language areas (Table 2), then saved for further use in correlation analysis. Patients who have hemispheric dominancy for language ipsilateral to the side of epilepsy (typical for left-TLE, atypical for right-TLE) were accepted to be at high risk for epilepsy surgery regarding possible postoperative language deficits. Plang, LI, nLI scores and patients at risk are shown in Table 2. 

### 3.2. Analysis of resting state-fMRI and calculation of functional connectivities

**Figure 4:**
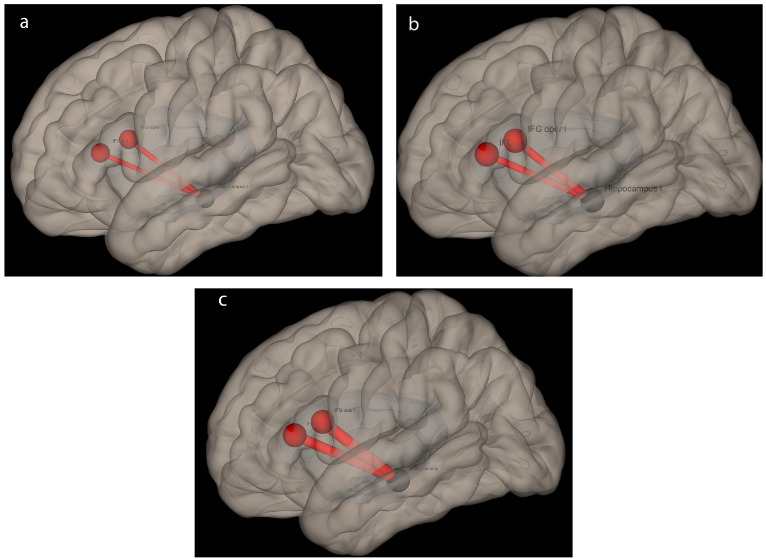
Functional connectivity analysis demonstrating intrahemispheric connections seeding from left hippocampus to language networks (CONN toolbox, SPM). Strength of connectivity slightly increases from left-TLE (A) to control (B) and right-TLE (C) subjects, respectively.” is more suitable.

Functional connectivity analysis was performed with rs-fMRI data in all subjects. FC values were calculated between hippocampal seeds and selected target ROIs (pars opercularis and triangularis, Broca) in both hemispheres with ROI-ROI analysis (Figures 4A–4C). FC values (β values) were extracted on a single subject basis and shown in Table 3.

**Table 3:** Functional connectivity results of participants.

Subject No.	Left hippocampal seed		Right hippocampal seed	
	Left pars opercularis	Left pars triangularis	Right pars opercularis	Right pars triangularis
1	–0.091	0.113	0.270	–0.042
2	–0.142	0.135	–0.150	0.004
3	0.141	0.118	0.140	0.021
4	0.026	0.134	–0.296	–0.052
5	0.001	0.084	–0.214	–0.032
6	–0.026	0.029	–0.294	0.001
7	–0.004	0.241	–0.239	–0.303
8	–0.359	–0.347	–0.299	–0.112
9	–0.338	–0.295	–0.266	–0.255
10	–0.088	–0.016	0.051	–0.021
11	0.076	–0.111	–0.201	–0.381
12	–0.048	–0.151	–0.353	–0.250
13	0.030	–0.024	–0.467	–0.236
14	0.092	0.146	–0.106	–0.201
15	–0.107	0.163	–0.073	–0.241
16	0.193	0.174	–0.016	0.027
17	–0.221	–0.164	–0.419	0.019
18	–0.140	0.192	0.003	0.125
19	–0.102	0.024	0.068	0.205
20	–0.084	0.038	0.172	–0.180
21	–0.023	–0.161	–0.504	–0.408
22	–0.087	0.001	0.080	–0.136
23	0.133	0.026	–0.031	–0.221
24	–0.340	0.298	–0.164	–0.022
25	0.225	0.303	–0.144	–0.080
26	0.166	0.175	0.020	0.214
27	0.047	0.100	–0.197	0.069
28	–0.038	0.084	0.066	–0.107
29	–0.056	0.069	0.004	–0.104
30	–0.271	0.202	–0.049	0.070
31	–0.213	0.064	–0.194	0.002
32	–0.073	0.058	–0.223	–0.212

Functional connectivity (FC) results are given in terms of beta values.

Among three groups there were no statistically significant difference found in terms of mean intrahemispheric FC values between left hippocampus and pars opercularis (F = 0.364; P = 0.186), left hippocampus and pars triangularis ( *x* *2* = 3.970, P = 0.137), right hippocampus and pars opercularis (F = 0.268; P = 0.767), right hippocampus and pars triangularis (F = 1.157; P = 0.329).

### 3.3. Correlation analysis between FC and nLI scores 

#### 3.3.1. Hippocampal seed, pars opercularis target

**Figure 5:**
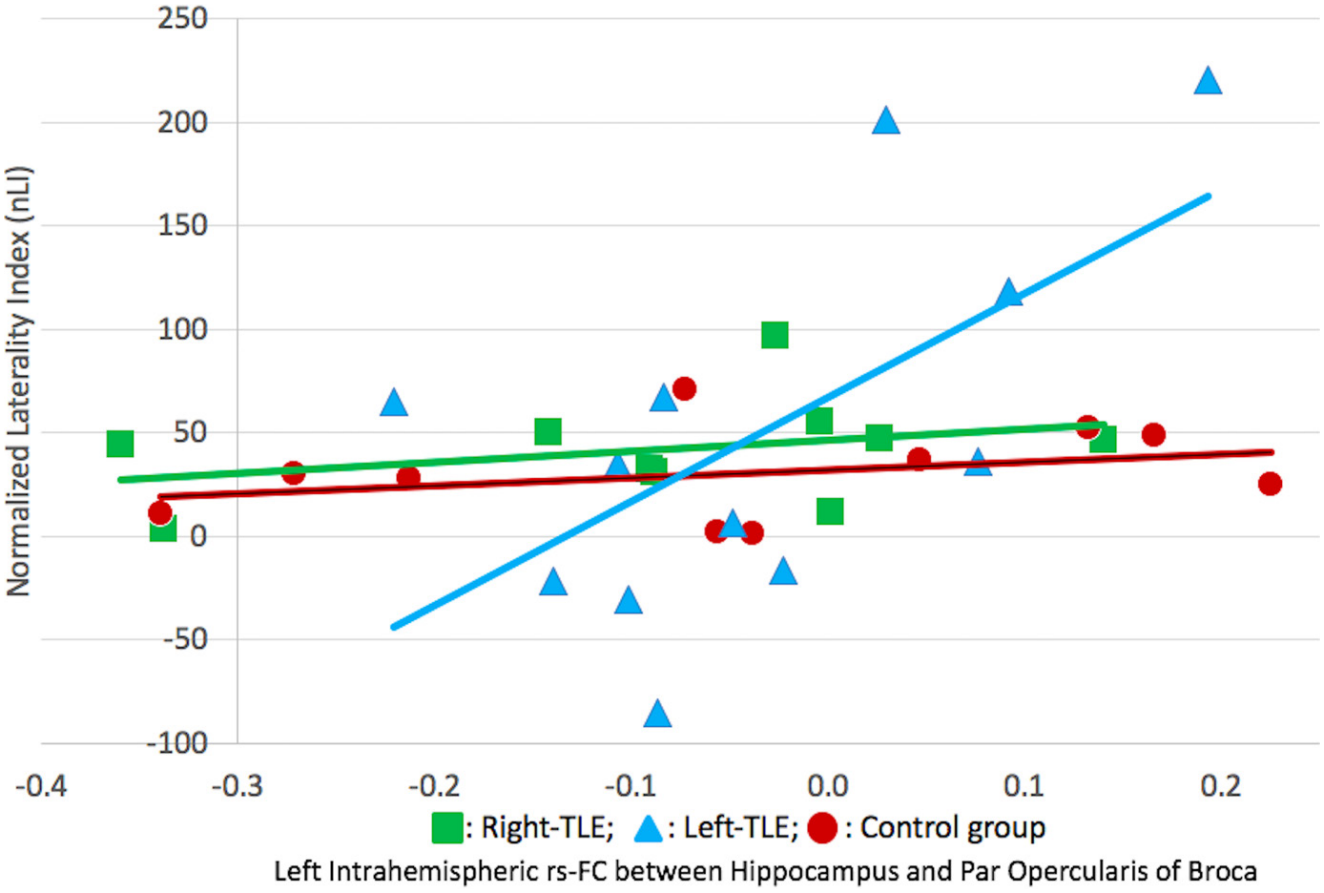
Chart showing correlation of nLI scores and rs-FC beta values in right-TLE, left-TLE and control group. Only statistically significant correlations are shown.

Left intrahemispheric FC values showed strong positive correlation with nLI scores in left-TLE, right-TLE patients and control group (Figure 5 and Table 4) ; moderate positive correlation in whole subjects (Table 4). There were no positive or negative significant correlation between right intrahemispheric FC values and nLI scores in left-TLE, right-TLE patients, control group and whole subjects (Table 4).

**Table 4:** Correlation analysis between FC and nLI scores.

Correlation with nLI	Right-TLE	Left-TLE	Control group	Whole subjects
(L)Hip.– (L)Oper.	r = 0.740 P = 0.014	r = 0.610 P = 0.035	r = 0.638 P = 0.047	Rho = 0.560 P = 0.001
(L)Hip.– (L)Tri.	Rho = 0.576 P = 0.082	r = 0.252 P = 0.430	Rho = –0.394 P = 0.260	Rho = 0.091 P = 0.622
(R)Hip.– (R)Oper.	Rho = –0.224 P = 0.533	r = –0.205 P = 0.523	r = –0.374 P = 0.288	Rho = –0.209 P = 0.252
(R)Hip.– (R)Tri.	Rho = 0.200 P = 0.580	r = –0.042 P = 0.896	r = –0.081 P = 0.823	Rho = –0.020 P = 0.914

FC: Functional connectivity; nLI: Normalized lateralization index; L: Left; R:Right; Hip: Hippocampus; Oper: Pars opercularis; Tri: Pars triangularis.

####  3.3.2. Hippocampal seed, pars triangularis target

There was no significant correlation between left and right intrahemispheric FC values and nLI scores in left-TLE, right-TLE patients, control group and whole subjects (Table 4).

Six right-TLE, five left-TLE patients were successfully operated at the time of this study. Anterior temporal lobectomy with amygdalahippocampectomy was the standard surgical approach applied in all cases. None of the patients had prominent language deficit and discharged from hospital for long term follow-up. 

## 4. Discussion

The questions we aimed to answer was the demonstration of intrahemispheric hippocampal-lingual functional connectivity and hippocampal lateralization for language with rs-fMRI. Our study results confirmed the existence of hippocampus-lingual functional network. This network was correlating well with lateralization of language function in the left hemisphere in both temporal lobe epilepsy patients and healthy controls. 

Task based functional connectivity analysis clearly defined areas of language in each separate subject. Broca’s and Wernicke’s areas are well known parts of frontal and temporal lobes, and mainly responsible for main motor and sensory functions of language. They have been investigated even before clinical use of functional MRI techniques. Emerging evaluation [23] of language functions have rose questions about consistency of classical Geschwind’s theory [24], but Broca located at IFG of the dominant hemisphere is currently the main consistent region of language that can be defined with functional MRI experiments. Pars opercularis and pars triangularis of Broca, represented by Brodmann’s cytoarchitectonic areas (BA) 44 and 45, are also responsible for complex cognitive functions including naming, phonologic, syntactic and semantic processes [25]. Therefore, we used these areas and their nondominant hemispheric mirrors to define ROIs representative of language networks. All participants successfully presented task-based activations at Broca’s region and its mirror on the right hemisphere.

Brain plasticity allows some recovery of lost cognitive functions after trauma or degenerative processes especially at younger ages. Left hemispheric cortical architecture that’s responsible from language may become disrupted in epilepsy patients [1]. Varying with the extent and timing of insult, plasticity leads to reorganization of language functions in TLE patients [5, 6, 26, 27]. We observed higher rates of atypical dominancy and lower mean LI scores in left-TLE patients compared to right-TLE patients and healthy controls. Highest mean LI score was observed in right-TLE patients which is actually an expected outcome. Since left hemisphere is dominant in ~95% of the human population, an epileptogenic insult in right hemispheric analogue of language regions would cause an additional increase in connections of left hemispheric language regions, which in turn will make the LI score higher in favour of typical dominancy. These results were compatible with previous fMRI and WADA studies; rate of atypical hemispheric dominancy in left-TLE patients were found 42.7% (Zatorre et al. [28]), 25% (Adcock et al. [25]), 38% (Brazdil et al. [6]). Doucet et al. [29] also found highest LI score in right-TLE patients. They also found only 4% atypical dominancy in their fMRI study but they stated that this could be resulted from the study group, having 23 years mean age of seizure onset. Duration of seizures and age at seizure onset are very important factors effecting language development processes. Hemispherectomy studies [30] and studies with pediatric stroke patients [31] have shown that hemispheric dominancy for language establishes around the age of 5, however older children and adults may also show varying levels of plasticity [25]. Higher ages of seizure onset may decrease the possibility of functional reorganization [27,32]. Mean age of onset was 14.2 years, which’s lower than the aforementioned study. Further studies in larger group of patients with different age of seizure onsets may define more precise functional connectivity changes. Nevertheless, our findings support the interhemispheric reorganization theory occurring in left-TLE patients by continuous disruption of cortical architecture.

Functional connectivity analysis was performed on single subject basis. Since we wanted to make a comparison of laterality indices and FC, we primarily focused on single subject measures obtained from resting state fMRI data. While mean FC values slightly differ between groups, our results did not show any difference in both hemispheres among three groups reaching statistical significance. This result was actually unexpected because other studies investigating FC in TLE patients mostly define varying amounts of decreased total hemispheric connectivity in the affected hemisphere [1,9,29,33]. Because we wanted to clarify if FC measures of “hippocampal-lingual network are decreasing in the affected side of epilepsy, we did not consider atypical hemispheric dominancy but analysed groups of right and left TLE patients instead. To reveal intrahemispheric connectivity, we defined both hippocampi as seed regions in ROI to ROI analysis. Decreased functional connectivity in the affected hippocampus might be a logical expectation, moreover some authors declare that FC may paradoxically increase with some compensatory mechanisms on the non-dominant hemisphere. Considering intrahemispheric resting state functional connectivity in TLE patients; Pravatà et al. [34] presented no difference between controls and patients in the right hemisphere while Bettus et al. [33] presented increased FC in the hemisphere contralateral to the side of epilepsy. Our results may be partially explained with these studies’ results. 

Hippocampus is thought to have an import role in cognitive processing via its cortical reciprocal connections. Its malfunction causes cessation of not only memory but also other various functions including language. Furthermore, these findings may be reversible after epilepsy surgery supporting the view that whole hemispheric cognitive impairment results from epileptogenic activity of hippocampus [35,36]. Hippocampus contribute to language functions by semantic memory functions or direct acquisition of language [36]. Effect of hippocampal sclerosis on language processing was very well discussed in former studies [6,37,38]. The most commonly accepted theory is the spread of epileptic seizures from hippocampus to the ipsilateral hemisphere thus impairing the basal cortical network. This, in turn causes TLE patients to lose language functions even if they do not have morphological abnormality on the arcuate fasciculus, Broca’s and Wernicke’s regions. Normal development processes include left hippocampal interaction with left hemispheric language areas [39] giving the idea of hippocampal laterality just as hemispheric dominancy of language. There are plenty of studies mainly focused on connectivities between two hippocampi or atrophied hippocampus and whole hemisphere/brain or intra- and interhemispheric connectivity between language networks but hippocampal laterality and correlation of resting state FC with LI scores [29] has been underestimated up to now, being discussed only in few of them [40, 41]. 

Our results have shown significant correlation between laterality indices (nLI) and “hippocampal-lingual measures of resting state functional connectivity (pars opercularis targeted) in the left hemisphere in all subjects. With results of LI analysis, which showed highest mean scores in right-TLE patients, it can be commented that interhemispheric reorganization of language functions occurs in TLE patients and thus causes stronger “hippocampal-lingual FC in right-TLE patients and vice versa. In another words; higher left intrahemispheric hippocampus-lingual FC values may predict typical; lower values may predict atypical hemispheric dominancy for language. Contrary to pars opercularis, there was no statistically significant correlation found between hippocampus and pars triangularis region of Broca in left hemisphere. This may be explained with lesser contribution of pars triangularis to the language network, just like Bernal et al. [42], Brauer et al. [43], Brown et al. [44], Diehl et al. [45], Doucet et al. [29] stated in their study or our word generation task could have distinctly activated subregions of Broca in favour of pars opercularis region. Furthermore, right intrahemispheric FC values did not correlate with LI. These results support the hypothesis of exclusive contribution of left hippocampus to the language. Hemispheric dominancy of language seems to be directly related with the functional reserve of the left hemisphere in the right-handed population. Right hemispheric mirrors of language networks only come forward in cases of left hemispheric insult. Similarly Pereira et al. [1], Josse et al. [46] and Ellmore et al. [47] depicted a higher level of left hemispheric connectivity associated with hippocampus in language functions than the right side. 

However, there are some limitations of this study. First, sample size was relatively small to make precise assumptions in some parts of the study. Studies with larger sample sizes may provide additional information. Second; although we described and used nLI scores for correlation with FC values, classical LI scores were used for determination of hemispheric dominancy. There is no reliable cut-off value for positive nLI scores referring to the LI score ranging between 0 and 10, due to different Plang values subjects may have. In other words, one can estimate that negative nLI values always refer to atypical dominancy whereas low positive nLI scores may not always define atypical dominancy alone. Thirdly, although they are routinely performed to all patients within pre-surgical workup by means of institutional policy, our study lacks results of neuropsychological tests. Laterality index scores could have been matched in terms of hemispheric dominancy of language. 

This fMRI study revealed the left “hippocampal-lingual network in right handed individuals of TLE patients and healthy subjects putting forward the crucial organizational role of hippocampus and effects of its damage on the left hemispheric reserve for language function. This data may provide a better understanding of reorganization of cortical functions in surgical planning of patients with seizures. 

## Main points

· We observed higher rates of atypical dominancy and lower mean LI scores in left-TLE patients compared to right-TLE patients and healthy controls.

· Higher left intrahemispheric “hippocampal-lingual FC values may predict typical; lower values may predict atypical hemispheric dominancy for language.

· Our findings support the interhemispheric reorganization theory occurring in left-TLE patients by continuous disruption of cortical architecture.

· Hemispheric dominancy of language seems to be directly related with the functional reserve of the left hemisphere in the right-handed population.

## Acknowledgment/Disclaimers

This study was presented at the European Congress of Radiology (ECR), 1-4 March 2017, in Vienna, Austria. 

## Informed consent

This study is approved by the institutional ethics committe (decision number: 25901600-958). Written informed consent was received from the participants.

Supplementary Materials Click here for additional data file. Dedicated epilepsy protocol for MR imaging.

## References

[ref1] (2010). Asymmetrical hippocampal connectivity in mesial temporal lobe epilepsy: evidence from resting state fMRI. BMC Neuroscience.

[ref2] (1992). Epilepsy: the role of MR imaging. AJR American Journal of Roentgenology.

[ref3] (2016). MRI-negative temporal lobe epilepsy: A network disorder of neocortical connectivity. Neurology.

[ref4] (2000). Handedness and hemispheric language dominance in healthy humans. Brain.

[ref5] (2009). Atypical language lateralization in patients with left hippocampal sclerosis: does the hippocampus affect language lateralization?. Turkish Neurosurgery.

[ref6] (2005). Reorganization of language-related neuronal networks in patients with left temporal lobe epilepsy - an fMRI study. European Journal of Neurology.

[ref7] (2017). Roles of the Wada Test and Functional Magnetic Resonance Imaging in Identifying the Language-dominant Hemisphere among Patients with Gliomas Located near Speech Areas. Neurologia Medico-Chirurgica.

[ref8] (2014). Defining language networks from resting-state fMRI for surgical planning--a feasibility study. Human Brain Mapping.

[ref9] (2010). Role of resting state functional connectivity MRI in presurgical investigation of mesial temporal lobe epilepsy. Journal of Neurology, Neurosurgery, and Psychiatry.

[ref10] (2017). Effective connectivity in temporal lobe epilepsy with hippocampal sclerosis. Acta Neurologica Scandinavica.

[ref11] (1995). Visual learning on a selective reminding procedure and delayed recall in patients with temporal lobe epilepsy. Epilepsia.

[ref12] (1998). Right hippocampal contribution to visual memory: a presurgical and postsurgical study in patients with temporal lobe epilepsy. Journal of Neurology, Neurosurgery and Psychiatry.

[ref13] (1971). The assessment and analysis of handedness: the Edinburgh inventory. Neuropsychologia.

[ref14] (2012). E-Prime User’s Guide. 2012. Sharpsburg, PA; USA: Psychology Software Tools, Inc..

[ref15] (2006). Analysis of functional image analysis contest (FIAC) data with brainvoyager QX: From single-subject to cortically aligned group general linear model analysis and self-organizing group independent component analysis. Human Brain Mapping.

[ref16] (2008). Laterality index in functional MRI: methodological issues. Magnetic Resonance Imaging.

[ref17] (2008). Reproducibility of fMRI in the clinical setting: Implications for trial designs. NeuroImage.

[ref18] (2007). Reproducibility of activations in Broca area with two language tasks: a functional MR imaging study. AJNR American Journal of Neuroradiology.

[ref19] (2002). Reproducibility of fMRI-determined language lateralization in individual subjects. Brain and Language.

[ref20] (2016). Validity and reliability of four language mapping paradigms. NeuroImage: Clinical.

[ref21] (2012). Conn: a functional connectivity toolbox for correlated and anticorrelated brain networks. Brain Connectivity.

[ref22] (2006). FMRI Shows Atypical Language Lateralization in Pediatric Epilepsy Patients. Epilepsia.

[ref23] (2016). Broca and Wernicke are dead, or moving past the classic model of language neurobiology. Brain and Language.

[ref24] (1970). The organization of language and the brain. Science New Series Vol. 170 No. 3961. New York, NY, USA: American Association for the Advancement of Science.

[ref25] (2003). Quantitative fMRI assessment of the differences in lateralization of language-related brain activation in patients with temporal lobe epilepsy. Neuroimage.

[ref26] (2002). Late plasticity for language in a child’s non-dominant hemisphere: a pre- and post-surgery fMRI study. Brain.

[ref27] (2011). Language organization and reorganization in epilepsy. Neuropsychology Review.

[ref28] (1989). Perceptual asymmetry on the dichotic fused words test and cerebral speech lateralization determined by the carotid sodium amytal test. Neuropsychologia.

[ref29] (2015). Resting-state functional connectivity predicts the strength of hemispheric lateralization for language processing in temporal lobe epilepsy and normals. Human Brain Mapping.

[ref30] (1997). Onset of speech after left hemispherectomy in a nine-year-old boy. Brain.

[ref31] (2017). The time window for successful right-hemispheric language reorganization in children. European Journal of Paediatric Neurology.

[ref32] (1999). Language dominance in neurologically normal and epilepsy subjects: a functional MRI study. Brain.

[ref33] (2009). Decreased basal fMRI functional connectivity in epileptogenic networks and contralateral compensatory mechanisms. Human Brain Mapping.

[ref34] (2011). Functional connectivity MR imaging of the language network in patients with drug-resistant epilepsy. AJNR American Journal of Neuroradiology.

[ref35] (2004). ILAE Commission Report. Mesial temporal lobe epilepsy with hippocampal sclerosis. Epilepsia.

[ref36] (2006). Left hippocampal pathology is associated with atypical language lateralization in patients with focal epilepsy. Brain.

[ref37] (2003). Atypical hemispheric language dominance in left temporal lobe epilepsy as a result of the reorganization of language functions. Epilepsy & Behavior.

[ref38] (2008). Functional MRI evidence for language plasticity in adult epileptic patients: preliminary results. Neuropsychiatric Disease and Treatment.

[ref39] (2002). Functional neuroimaging of speech perception in infants. Science.

[ref40] (2014). Structural connectivity differences in left and right temporal lobe epilepsy. Neuroimage.

[ref41] (2015). Distinct functional connectivity of the hippocampus during semantic and phonemic fluency. Neuropsychologia.

[ref42] (2010). The connectivity of the superior longitudinal fasciculus: a tractography DTI study. Magnetic Resonance Imaging.

[ref43] (2011). Neuroanatomical prerequisites for language functions in the maturing brain. Cerebral Cortex.

[ref44] (2014). Evaluating the arcuate fasciculus with combined diffusion-weighted MRI tractography and electrocorticography. Human Brain Mapping.

[ref45] (2010). Cortical stimulation for language mapping in focal epilepsy: correlations with tractography of the arcuate fasciculus. Epilepsia.

[ref46] (2009). Predicting language lateralization from gray matter. Journal of Neuroscience.

[ref47] (2010). Temporal lobe white matter asymmetry and language laterality in epilepsy patients. Neuroimage.

