# Supplementary material for: Correlation of language lateralization with resting state hippocampal connectivity in temporal lobe epilepsy patients
Source: Turk J Med Sci. 2020 Aug 26;50(5):1350–63. doi: 10.3906/sag-2001-29 (PMC7491274; doi:10.3906/sag-2001-29)
Supplement: Supplementary file 1 — Supplementary Materials [file turkjmedsci-50-1350-sup001.pdf]

Parameters / Sequences	TE (ms)	TR (ms)	Slice Thickness (mm)	FOV (mm)	Slice number
T1w axial	9	360	5	220*320	20
T1w sagittal	9	400	5	240*240	20
T2w axial	101	3500	3	230*230	20
FLAIR axial	90	9000	5	208*230	20
FLAIR coronal oblique	90	9000	3	180*230	20
T1-IR coronal oblique	380	3000	1	190*220	104
DWI axial (b1000)	90	9800	3	220*220	40
T1w sagittal	2,5	1900	1	250*250	176

***Note-** Coronal oblique sequences were acquired perpendicular to the long axis of hippocampus
